# The Impact of Nursing Staffs’ Working Conditions on the Quality of Care Received by Older Adults in Long-Term Residential Care Facilities: A Systematic Review of Interventional and Observational Studies

**DOI:** 10.3390/geriatrics7010006

**Published:** 2021-12-28

**Authors:** Elodie Perruchoud, Rafaël Weissbrodt, Henk Verloo, Claude-Alexandre Fournier, Audrey Genolet, Joëlle Rosselet Amoussou, Stéphanie Hannart

**Affiliations:** 1 Department of Nursing Sciences, School of Health Sciences, HES-SO Valais/Wallis, University of Applied Sciences and Arts Western Switzerland, Chemin de l’Agasse 5, CH-1950 Sion, Switzerland; rafael.weissbrodt@hevs.ch (R.W.); henk.verloo@hevs.ch (H.V.); calexandre.fournier@hevs.ch (C.-A.F.); audrey.genolet@hevs.ch (A.G.); stephanie.hannart-oppliger@hevs.ch (S.H.); 2 Service of Old Age Psychiatry, Department of Psychiatry, Lausanne University Hospital, Route de Cery 60, CH-1008 Lausanne, Switzerland; 3 Psychiatry Library, Education and Research Department, Lausanne University Hospital and University of Lausanne, Site de Cery, CH-1008 Lausanne, Switzerland; joelle.rosselet@chuv.ch

**Keywords:** health care, older adults, long-term residential care facilities, working conditions, quality, nursing staff

## Abstract

Background: Little documentation exists on relationships between long-term residential care facilities (LTRCFs), staff working conditions and residents’ quality of care (QoC). Supporting evidence is weak because most studies examining this employ cross-sectional designs. Methods: Systematic searches of twelve bibliographic databases sought experimental and longitudinal studies, published up to May 2021, focusing on LTRCF nursing staff’s working conditions and the QoC they provided to older adults. Results: Of the 3577 articles identified, 159 were read entirely, and 11 were retained for inclusion. Higher nursing staff hours worked per resident per day (HPRD) were associated with significant reductions in pressure sores and urinary tract infections. Overall staff qualification levels and numbers of RNs had significant positive influences on QoC. Conclusions: To the best of our knowledge, this systematic review is the first to combine cohort studies with a quasi-experimental study to explore associations between LTRCF nursing staff’s working conditions and older adult residents’ QoC. Human factors (including HPRD, staff turnover, skill mix, staff ratios) and the specific working contribution of RNs had overwhelmingly significant influences on QoC. It seems essential that LTRCF supervisory and decision-making bodies should promote optimal working conditions for nursing staff because these have such a direct impact on residents’ QoC.

## 1. Introduction

A challenge facing most modern societies, caused by ageing populations, is the increasing demand on health and care services [1]. Ageing increases the risks of developing multiple chronic conditions, leading to patients with complex long-term care needs [2]. Across European countries, ageing populations are creating a growing demand for health care staff, particularly nursing specialists in geriatric or psychogeriatric care, to care for older adults with complex, age-related, somatic and psychopathological conditions [3,4]. Thus, it seems likely that a major part of the nursing and medical care required for this population will be redirected from hospitals to home health care or assisted living teams, intensifying the need for highly-specialized geriatric care [5]. The lack of highly qualified geriatric and psychogeriatric health care professionals poses a critical threat to patient safety and the ability to provide evidence-based care [6]. This is consistent with evidence from other health care domains such as hospitals, where several studies have pointed out that higher proportions of nurses with bachelor’s degrees are related to reductions in patient mortality [7,8]. The current global demographic transition ensures that there will be significant demand for certified nursing assistants (CNAs), licensed practical nurses (LPNs), and registered nurses (RNs) working in geriatrics and psychogeriatric care in long-term residential care facilities (LTRCFs) for many years to come [9]. LTRCFs are important components of the increasingly complex health care systems that are being stretched by growing demands for services [10].

Poor quality nursing care in LTRCFs has been associated with inadequate staff working conditions because working conditions are presumed to affect quality of care (QoC) and the lives of nursing home residents [11,12]. The relationship between staff working conditions and the QoC in LTRCFs has received considerable attention in recent years, but reviews of those studies revealed weak levels of evidence [13,14] These investigations were based mostly on cross-sectional studies and were possibly biased because the confounding factors that affect nursing-home quality correlated with the explanatory variables used in the studies [15]; the study designs may account for the weak associations found [11,14]. More evidence is needed, especially from experimental and longitudinal studies.

The present work aims to review recent experimental and longitudinal studies focusing on nursing staff’s working conditions and the QoC provided by CNAs, LPNs, and RNs to older adults in LTRCFs. Our research question was: Is there an association between nursing staff’s working conditions and the QoC provided to older adults in LTRCFs? Most nursing staff are engaged in physically and psychologically demanding work, juggling multiple professional and family responsibilities [16]. It is not surprising that nursing staff turnover in LTRCFs is consistently high [11,17]. In addition, nursing staff often experience physical ailments such as musculoskeletal disorders and mental health problems such as burnout, emotional exhaustion, or distress [17,18,19]. Different staff, at different levels in the hierarchy, face diverse working conditions in LTRCFs. CNAs, LPNs, and RNs perform different tasks and take on different professional roles and responsibilities in residential care. For example, CNAs provide direct primary care to residents and assist them in their daily activities. LPNs work with RNs to assess, coordinate, and implement residents’ nursing care needs. RNs mostly develop residents’ care plans, implement treatments, perform assessments and evaluations, and oversee the tasks of LPNs and CNAs. Previous studies have reported that CNAs have less control over their working conditions than do RNs, but there have also been differing results on whether CNAs and RNs face different psychological demands [20,21,22]. Poorly adapted working conditions cause a high level of stress among CNAs, and previous studies have reported relationships with such work organization factors as for-profit ownership, managerial pressure, and a lack of pay increases [22,23,24].

However, only sporadic attention has been paid to whether the working conditions of different types of nursing staff affect nursing-home residents’ safety and their QoC [25]—elements that merit close attention from researchers and policymakers [26,27]. Many factors influence the QoC provided to residents: some are internal to a nursing home’s organization, such as staffing levels and characteristics, the nursing staff’s level of education and training, job satisfaction and staff turnover, salaries and benefits, and the management and organizational atmosphere. Others are external to the facility itself, such as regulations, reimbursement policies, incentives, and excessive demand for services [6].

Previous studies have demonstrated that higher staffing levels and a lower turnover of RNs are related to functional institutional improvements. They also found that low staffing levels in nursing homes with very dependent residents were associated with a reduced likelihood of improvements to residents’ health. Some evidence has been found that staff-to-resident ratios have a significant impact on residents’ health outcomes [6]. A 2011 study by Castle et al. [28] reported that more nursing staff, a greater professional staff mix, and lower nursing staff turnover in LTRCFs were significantly associated with less use of physical restraint, fewer residents requiring a urinary catheter, fewer pressure sores, and better pain management.

The impact of staff working conditions on the health outcomes of LTRCF residents has been studied since the development of Karasek’s demand–control–support model [29,30]. The model emphasizes that employees experiencing sub-optimal working conditions—high job demands, low job control, and low social support—are considered to be in highly strenuous jobs, which is associated with an increased risk of physical and mental deterioration, which in turn influences the QoC provided to LTRCF residents [23,31]. Managers in LTRCFs should consider improvements in working conditions to be part of their overall strategy for maintaining or improving their nursing staff’s health outcomes. Interventions might include reducing intense workloads and understaffing [32], rethinking the complex care required for cognitively impaired residents [19], reducing workplace assaults and violence [33], and improving irregular work schedules [34]. Some evidence suggests that unfavorable working conditions, such as a lack of management support, autonomy, and professional recognition [35], together with demanding work schedules, have observable negative effects on the care provided to older adults in LTRCFs [36].

## 2. Materials and Methods

The present work followed the Joanna Briggs Institute’s guidelines for systematic reviews and was based on the Preferred Reporting Items for Systematic Reviews and Meta-Analyses (PRISMA) recommendations [37]. Systematic review registration: PROSPERO 2021: CRD42021226656

### 2.1. Types of Studies

This review included publications on primary research if they (1) examined the relationship between nursing staff’s working conditions and the QoC received by nursing-home residents, (2) only included LTRCFs, and (3) were original research articles describing observational, longitudinal, or experimental quantitative studies. There were no restrictions on language, country of origin, or publication date.

### 2.2. Types of Participants

The types of staff who had participated in the studies were certified nursing assistants (CNAs), licensed practical nurses (LPNs), and registered nurses (RNs). CNAs are defined as professional health care workers who have had several months of health care training and who provide direct care to residents, under the supervision of LPNs or RNs. LPNs are defined as professional health care workers who have completed a two-to-three-year health care training program and who provide support services to RNs. RNs are defined as professional health care workers who have completed a three-to-four-year health care studies program, obtained a bachelor’s degree or equivalent, and who perform many basic and advanced nursing tasks.

### 2.3. Factors of Interest

Factors of interest were any individual or organizational variables associated with staff working conditions. The European Foundation for the Improvement of Living and Working Conditions describes working conditions as “the conditions in and under which work is performed. A working condition is a characteristic or a combination of characteristics of work that can be modified and improved” [38]. According to the International Labour Organization, working conditions cover a broad range of topics and issues, from working time (hours of work, rest periods, and work schedules) to remuneration, as well as the physical conditions and mental demands that exist in the workplace [39].

In the health field, the World Health Organization defines several key components of working conditions for nursing staff, including working hours, shift work, workload, staffing levels, and clinical rotation [40]. Nurse staffing levels are a major factor in nurses’ working conditions and are directly related to the QoC [41]. According to the International Council of Nurses, safe nurse staffing levels require that an appropriate number of nurses are available at all times, across the continuum of care and with a suitable mix of education, skills, and experience to ensure that patient care needs are met and that the working environment and conditions support staff to deliver quality care [41]. Inadequate or insufficient nurse staffing levels increase the risk of care being compromised and have negative impacts on staff health and well-being [41].

### 2.4. Outcomes of Interest

The outcomes of interest were whether nursing staff’s working conditions had an impact on the QoC received by LTRCF residents and which QoC outcomes were influenced by nursing staff’s working conditions.

The Institute of Medicine defined QoC as “the degree to which health services for individuals and populations increase the likelihood of desired health outcomes and are consistent with current professional knowledge.” This led to a definition of quality that appears as lists of quality indicators, which are outcomes that represent high standards [42].

Regarding the QoC outcomes in this review, we will distinguish between clinical outcomes (e.g., pressure ulcers, different types of infections, health status, weight loss) and process-related outcomes (e.g., deficiencies linked to the QoC, indicators of the QoC, avoidable hospitalization).

### 2.5. Search Strategy for the Identification of Relevant Studies

The search was conducted in May 2020, supported by a medical librarian, in the following bibliographic databases: Embase.com, Medline Ovid, PubMed (not medline[sb]), CINAHL EBSCO, APA PsycINFO Ovid, Cochrane Library Wiley, Web of Science Core Collection, and the Joanna Briggs Institute (JBI) EBP Database OvidSP. All searches were conducted without language or date restrictions. The detailed search strategy is available in Appendix A. The bibliographies of relevant articles were examined, and in January 2021 additional searches were performed in Google Scholar, SantéPsy (Ascodocpsy), Dart Europe, ProQuest Dissertations & Theses A&I, and OpenGrey. Finally, a search for references citing key articles (i.e., forward citation chasing) was performed in the Web of Science Core collection.

### 2.6. Study Screening and Data Extraction

The retrieved articles were managed in an Endnote library (version X9). Three researchers (AG, EP, and RW) independently screened titles and abstracts for relevance. After reaching a consensus on the results of their independent screening processes, the full-text articles of potentially relevant studies were obtained. The same team members independently screened the full-text articles, labeling them ‘include’ or ‘exclude’ from the review. They discussed and resolved any disagreements so as to reach a consensus about the final list of included studies.

Two researchers (EP and SH) extracted data from all the included articles using a standardized form specifically developed for this review. The data collected included publication type, context and setting, study aims, methodology, independent variables of working conditions, covariates, study findings, potential study limitations, and study recommendations. The data extracted were discussed within the research team.

### 2.7. Methodological Quality

The methodological quality of included studies was assessed using the Newcastle–Ottawa Scale for Assessing the Quality of Non-Randomized Studies in Meta-Analysis [37]. This consists of eight items covering three domains: selection (representativeness of the cohort), comparability (controlling for confounders), and outcomes (assessment and follow-up). Two researchers (EP and SH) independently rated each included study’s quality on a scale from 0 stars to 9 stars, classifying them into groups of low (< 6 stars), moderate (6–7 stars), or high (8–9 stars) quality [37]. Researchers discussed any disagreements to reach consensus.

We used the validated Robins-I tool for assessing the risk of bias in non-randomized studies of interventions (NRSIs) [38]. This tool covers two dimensions and seven domains through which bias might be introduced into an NRSI: (i) pre-intervention and at-intervention bias (due to confounding, the selection of study participants, or the classification of the intervention), and (ii) post-intervention bias (due to missing data, deviations from intended interventions, bias in the measurement of outcomes, or bias in the selection of the reported result) [38]. Any disagreements in quality assessments were resolved through discussion.

## 3. Results

### 3.1. Search Strategy Results

**Figure 1:**
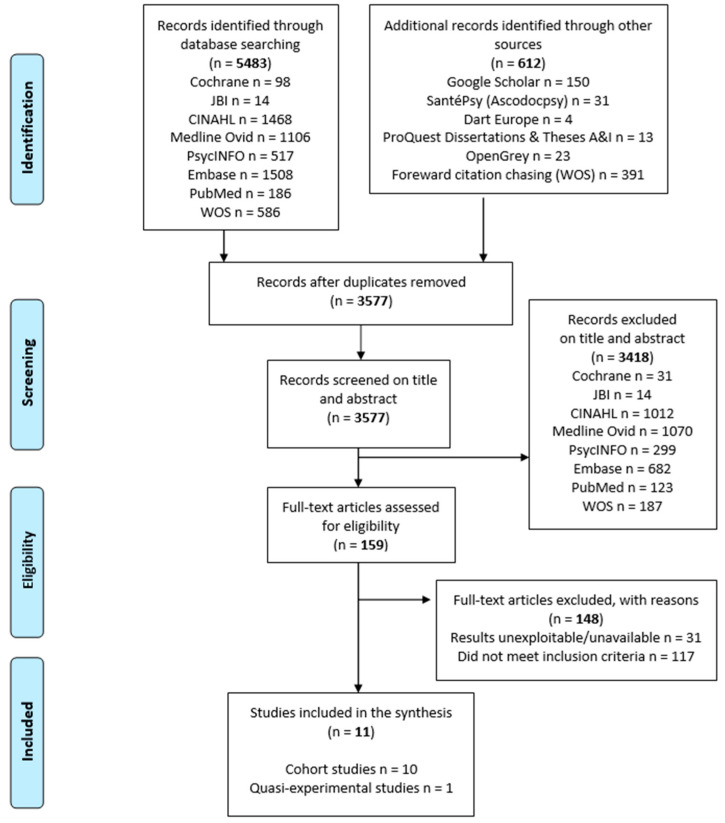
Flow diagram summarizing the results of our search strategy based on the PRISMA recommendations [37].

Our strategy of searching bibliographic databases retrieved a total of 3577 articles after the elimination of duplicates. Based on their titles and abstracts, 159 articles were retained as potentially eligible, and their entire texts were evaluated. In the end, only 11 articles met our selection criteria and were included: 10 cohort studies and one quasi-experimental interventional study (Figure 1).

### 3.2. Characteristics of Studies, Participants, and Institutions

The eleven included studies were carried out in four countries (Germany, China, South Korea, and the USA), across three continents (Europe *n* = 2, Asia *n* = 3, and North America *n* = 6), and published between 1977 and 2018. Ten were cohort studies [40,41,42,43,44,45,46,47,48,49], and one was a quasi-experimental interventional study [50] (Table 1).

Four of the cohort studies were retrospective and based on 406,632 observations taken from reports and databases (M = 135,544; SD = 228,341; range = 2493 to 399,206) covering 3173 nursing homes (M = 793; SD = 577; range = 45 to 1366) over periods ranging from 2.75 to 5 years (M = 3.94; SD = 0.92) [43,46,49,56].

Six cohort studies involved 64,139 residents (M = 18,325; SD = 23,890; range = 346 to 46,044) in 925 nursing homes (M = 264; SD = 318; range = 4 to 534) over periods ranging from 0.33 to 9 years (M = 4.74; SD = 5.68) [50,53,55,58,59,60]. Three studies reported on residents’ ages, which ranged from 65 to 100 years old (M = 76.4; SD = 7.50), with 55.4% men (SD = 38.82) and 44.6% women (SD = 38.82) [50,53,59].

Only the study by Temkin et al. [58] documented the numbers of professionals participating (*n* = 7418), of whom 50% were CNAs, 19% were LPNs, and 13% were RNs, while 9% were therapists and 9% were other professionals. The other studies included nursing professionals by number and type of qualification [43,46,49,50,53,55,56,59,60].

The quasi-experimental interventional study by Burgio et al. [62] compared two models of professional caregiver staffing numbers: a first group of 104 residents cared for by a permanent staff of 91 CNAs and a second group of 192 residents cared for by a rotating staff of 178 CNAs. There were no major significant differences in the characteristics of the residents and CNAs available for comparison except for the permanent or rotating staffing models.

Given the heterogeneity of the data included in our selected studies, it was impossible to carry out a meta-analysis of their groups or subgroups.

### 3.3. Methodological Quality of the Studies

The overall methodological quality of the cohort studies included in this review was poor-to-moderate (Table 2).

None of our included studies was rated as having a high total methodological quality score (eight to nine stars): five were given a moderate score (six to seven stars) [43,46,50,53,55], and five scored as having poor methodological quality (fewer than six stars) [49,56,58,59,60]. The different domains of evaluation used on the quasi-experimental interventional study by Burgio et al. [62] were scored as having methodological quality ranging from a weak to moderate risk of bias, with an overall risk of bias classed as moderate.

### 3.4. Description of the Staffing Levels in the Studies

The authors of the selected studies used different means of defining staffing levels, generally referring to national laws and standards. Several authors collected data on HPRD, which is calculated by dividing the total number of hours worked by all the professional caregivers in an LTRCF over 24 h by the number of residents [43,46,49,53,56,58] (Table 3).

Konetzka et al. [49] looked at RNs’ HPRD and the staffing skill mix, defined as RNs’ proportion of the total hours of care given by RNs, LPNs, and CNAs. RNs’ mean HPRD was 0.35 (SD = 0.22), and their mean proportion of the skill mix was 0.12 (SD = 0.06) [49]. Shin et al. [56] looked at the HPRD of RNs, CNAs, and qualified care workers (QCWs), who, according to some authors, correspond to the USA’s definition of CNAs [56].

Yoon et al. [59] defined the nurse staffing level as the number of nursing staff (RNs and LPNs) per 100 beds. They also looked at the ratio of RNs, defining that as the ratio of RNs to all nursing staff. The mean nurse staffing level was 15.58 nurses per 100 beds, and the ratio of RNs was 0.56, suggesting that RNs made up about half of the total nursing staff [59].

Kwong et al. [50] examined the number of full-time CNAs per 100 residents. Across the four LTRCFs in their sample, the mean was 14.85 (SD = 9.85) [50].

Finally, German law states that RNs must make up at least 50% of the nursing staff in LTRCFs [55,60]. Zimmerman et al. [60] defined staffing levels as the ratios between the number of full-time equivalents (FTEs) for each type of caregiver and the residents. The proportion of RNs in their sample of LTRCFs ranged from 31.6% to 90.6%, with a mean of 56.7% [60]. Popp et al. [55] considered the proportion of FTE staff who were active, qualified personnel caring for residents. The proportion of qualified personnel in each different establishment ranged from 46% to 75%, with a mean of 58.1% [55].

### 3.5. Clinical Outcomes

Four studies examined associations between working conditions and the development of pressure ulcers [49,50,55,58] (Table 4), with three of them evaluating relationships between nurse staffing levels or their HPRD and the development of pressure ulcers [49,50,58]. Konetzka et al. [49] indicated that an increase in RNs’ HPRD significantly reduced the probability of developing pressure ulcers (*p* = 0.01). The total number of hours worked by care staff, i.e., the combined hours of RNs, LPNs, and CNAs, did not have a statistically significant influence on the development of pressure ulcers in the studies by Konetzka et al. [49] (*p* > 0.05) or Temkin et al. [58] (*p* = 0.61). Two studies looked at nursing staff’s levels of qualification and the development of pressure ulcers [50,55]. In the study by Kwong et al. [50], residents in nursing homes where there were RNs had a significant 26% lower probability of developing pressure ulcers (*p* ≤ 0.001). Finally, one study examined the relationship between the prevalence of pressure ulcers and teamwork and several managerial aspects [58]. Temkin et al. [58] revealed a significant reduction in the probability of developing pressure ulcers in LTRCFs displaying better team cohesion (*p* = 0.03) and greater nursing autonomy (*p* = 0.03).

Three studies investigated associations between working conditions and residents’ urinary function or the development of urinary infections [49,58,59] (Table 4). In the study by Konetzka et al., increases in RNs’ HPRD and the combined number of hours worked by RNs, LPNs, and CNAs led to statistically significant reductions in the probability of developing urinary infections (*p* = 0.01) [49]. According to Yoon et al. [59], an increase in the standard deviation (0.19) of RN staffing levels led to a significant 80% increase in the probability of improved or stable urinary incontinence (*p* = 0.02). By contrast, the combined number of hours worked by RNs, LPNs, and CNAs in the study by Temkin et al. [58], and the combined staffing levels of RNs and LPNs per 100 beds in the study by Yoon et al. [59], had no statistically significant influence on the probability of urinary incontinence (*p* = 0.22) or the probability of improved or stable urinary incontinence (*p* = 0.37), respectively. A 0.23 increase in the standard deviation of the team cohesion score in the study by Temkin et al. [58], reduced the probability of incontinence by a statistically significant 7.6% (*p* < 0.001).

Linn et al. [53] evaluated associations between the respective numbers of hours worked by RNs, LPNs, and CNAs and nursing home residents’ health status over a period of six months. Only the number of hours worked by RNs had a significant positive influence on residents’ health status, as evidenced by the fact that the LTRCFs where RNs worked the most hours had lower mortality rates, less deterioration in residents’ health status, and fewer hospital admissions (*p* < 0.05) (Table 4).

Zimmermann et al. [60] examined associations between the number of residents per RN, the number of residents per CNA, and residents’ weight loss. One extra resident per RN significantly increased the probability of residents losing weight by 2.3 times (*p* ≤ 0.01), whereas one extra resident per CNA had no statistically significant influence on weight loss (*p* ≥ 0.05) (Table 4).

Structural and organizational factors, such as bed occupancy rates, nursing home size, whether the institution was private or public, whether it was in an urban or rural location, and whether nursing staff were assigned to residents on a permanent or rotating basis, had no statistically significant influence on the development of pressure ulcers, urinary infections, changes in urinary function, health status, or weight loss among residents [49,53,58,59,60] (Table 4).

Finally, the quasi-experimental interventional study by Burgio et al. [62] sought to differentiate between the effects on residents of having permanent or rotating CNA staffing assignments, especially by looking at the spoken interactions between caregivers and residents, as well as at residents’ disruptive behaviors, hygiene, appearance, and their self-perceived emotions. This study only found a statistically significant difference between staffing systems for the quality of care [62]. Higher scores were also noted for residents’ personal appearance and hygiene under the permanent CNA staffing model (*p* = 0.04) [62] (Table 5).

### 3.6. Process-Related Outcomes

Two studies investigated associations between the HPRD of RNs, LPNs, and CNAs and the number of deficiencies linked to care or the quality of care (QoC) declared in each LTRCF [43,46] (Table 4). Kim et al. [46] reported that an increase in the total number of nursing hours worked (RN plus LPN plus CNA hours), significantly reduced the total number of deficiencies (*p* < 0.001), the number of deficiencies linked to the QoC (*p* < 0.001), and the number of severe deficiencies altering the safety of care (*p* < 0.05). By looking at the hours worked by each professional group, a significant reduction in the total number of deficiencies and the number of deficiencies linked to the QoC was observed as more hours were worked by RNs (*p* < 0.001 and *p* < 0.01, respectively), by LPNs (*p* < 0.001), and by CNAs (*p* < 0.001) [46]. The number of hours worked by RNs and LPNs had no statistically significant effect on the number of severe deficiencies altering the safety of care (*p* > 0.05), whereas an increase in the number of hours worked by CNAs significantly reduced the number of severe deficiencies (*p* < 0.05) [46]. However, Hyer et al. [43] considered the joint influence of the hours worked by LPNs and RNs together (and separately from those worked by CNAs) on the total number of declared deficiencies and the number of deficiencies linked to the QoC. It highlighted, on the contrary, that the only variable associated with a statistically significant reduction in deficiencies linked to the QoC was an increase in the number of hours worked by CNAs (*p* = 0.02).

Finally, Shin et al. [56] looked at the associations between the HPRDs of RNs, LPNs, and CNAs, the skill mix (the ratio of RNs to LPNs, and the ratio of RNs to CNAs), staff turnover, and 15 other indicators of the QoC (the prevalence of falls, pressure sores, aggressive behavior, depression, cognitive decline, incontinence, urinary tract infection, weight loss, dehydration, tube feeding, bed rest, activities of daily living, residents’ range of motion, antidepressant or sleeping pill use, and the need for physical restraint). A one-hour increase in the HPRD of RNs was associated with a statistically significant 3.9% lower rate of depression among residents (*p* = 0.002), a 5.7% lower prevalence of bedridden residents (*p* = 0.05) and a 1.1% lower use of physical restraints (*p* = 0.02) [56]. Furthermore, LTRCFs employing more RNs than LPNs observed significantly lower levels of aggressive behavior (*p* = 0.03), depression (*p* = 0.02), weight loss (*p* = 0.03), and being bedridden among their residents (*p* = 0.04) [56]. A greater ratio of RNs to CNAs was significantly associated with residents suffering less weight loss (*p* = 0.05) [56]. Finally, a significant positive statistical relationship was observed between the administration of antidepressants and sleeping pills and RN staff turnover (*p* < 0.001) and LPN staff turnover (*p* = 0.02), whereas no significant associations were noted between CNA staff turnover and different indicators of QoC [56]. When RN staff turnover rose by 5.9%, the prevalence of residents taking antidepressants or sleeping pills rose by 27.2%, whereas when LPN staff turnover rose by 9.7%, the prevalence of residents taking antidepressants or sleeping pills rose by 18% [56] (Table 4).

## 4. Discussion

The present systematic review aimed to identify cohort and experimental studies exploring associations between the working conditions of nursing staff and the quality of care (QoC) received by older-adult residents living in LTRCFs. We identified and incorporated ten cohort studies and one quasi-experimental interventional study into our review, covering a total of 64,139 residents and 406,632 observations. These combined pieces of research helped us to distinguish the influence of nursing staff’s working conditions on two types of results: residents’ clinical outcomes and results linked to processes and care pathways.

Regarding residents’ clinical outcomes, higher overall rates of nursing staff’s total HPRD were associated with the significantly better prevention of poor clinical outcomes such as the development of pressure ulcers or urinary tract infections (UTIs). Specifically, the greater the number of hours worked by registered nurses (RNs) or the greater the number of RN staff employed, the greater the real positive impacts on the different clinical outcomes measured among residents, notably in preventing the development of pressure ulcers and UTIs, improving urinary function and general health status, and reducing hospital admissions and the mortality rate. However, this was not true for licensed practical nurses (LPNs) and certified nursing assistants (CNAs). The importance of nursing staff’s qualification levels was also observed because RNs’ specific skills and knowledge were associated with greater positive influences on preventing the development of pressure ulcers and UTIs and improving urinary function than were those of LPNs and CNAs. The number of residents cared for per member of the nursing staff was also an important factor because an increase of one resident per RN was associated with a significantly higher risk of weight loss among those residents. Certain organizational aspects, such as effective teamwork, good team cohesion, and more nursing autonomy, were associated with positive impacts on residents’ clinical outcomes. Other organizational factors, such as permanent or rotating staff assignments to residents or the LTRCF’s occupancy rate, had no influence on residents’ clinical outcomes, nor did certain structural factors such as the size of LTRCFs, whether they were privately or publicly run, and whether they were situated in urban or rural areas.

With regards to results linked to care processes, the importance of higher total HPRD for all nursing staff was also highlighted because this was favorably associated with lower numbers of deficiencies linked to care or to the QoC, as well as with lower numbers of severe deficiencies declared by LTRCFs. The QoC was ensured by RNs’ specific contributions to improving QoC indicators. Finally, faster staff turnover was associated with a significant negative impact on QoC indicators.

The present systematic review had some limitations. Despite a thorough literature search using recognized guidelines and recommendations on methodology, our review may have missed some studies which met all the selection criteria due to study search errors or investigator mistakes. Three of the studies selected used the Online Survey, Certification, and Reporting (OSCAR) database [65] to collect data on nursing staff’s HPRD and the structural characteristics of the LTRCFs participating. However, OSCAR’s accuracy and validity, in these studies, were somewhat contested. Indeed, nursing professionals’ HPRDs were only calculated over a two-week period, which may not have been adequately representative of their true HPRD over a longer timeframe. Eight studies evaluated residents’ clinical outcomes using data reported by nursing staff themselves, which creates a risk of bias. In addition, the selected studies predominantly used nursing staff’s HPRD as the independent variable of interest, which may have led to an over-representation of this variable compared to other factors influencing the QoC. It is also difficult to draw any conclusions on the influence of the structural characteristics of LTRCFs as most of the studies did not explore the direct impacts of those variables on the QoC; instead, they used them as control variables during statistical analyses. Furthermore, there was a lot of heterogeneity in the follow-up periods chosen by the different cohort studies, varying between four months and nine years. Moreover, none of the cohort studies was given a high score for the quality of its methodology: five were considered moderate and four were of poor methodological quality. The one quasi-experimental interventional study, for its part, had a moderate risk of bias. Finally, any generalization of the present findings should be made with caution as the LTRCFs studied were always representative of a particular region or country.

Overall, the present systematic review included ten cohort studies and one quasi-experimental interventional study examining large samples of LTRCFs, residents, and observations using accurate, valid measurement instruments. Furthermore, we used highly recommended methodological norms and guidelines, making our findings very reliable. To the best of our knowledge, no systematic reviews incorporating cohort and experimental studies have been published to date on how nursing staff’s working conditions affect the QoC received by older adults living in LTRCFs. Other systematic reviews on this interesting topic mainly drew together studies of a transversal design, potentially biased by the numerous confounding factors inherent in such designs. The present systematic review thus helps to provide a higher level of proof.

In view of the small number of experimental studies in our field of interest to date, there is a need for further interventional research on the impact of nursing staff’s working conditions on the QoC received by older adults living in LTRCFs. Providing safe, high-quality care is the primary objective of all health care institutions. With a view to attaining continuous improvements in quality and safety, more research data on the relationship between nursing staff’s working conditions and the QoC provided to residents would help to support recommendations to health care managers, supervisors, political decision-makers, and other stakeholders involved in long-term care. More data would help to establish better working conditions, notably with a view to defining a standard minimum level of nursing staff necessary to ensure optimal care for older adults living in LTRCFs.

Finally, most of the studies identified in this systematic review underlined the tendency for LTRCFs to reduce their numbers of RNs and hire more LPNs and CNAs in order to reduce the overall costs of nursing personnel. However, most of these studies also pointed out the specific contributions of RNs in maintaining and improving the QoC. Thus, particular attention should be given to the presence of enough RNs in an LTRCF to supervise and monitor the care dispensed by their LPN and CNA colleagues. This approach will enable staff to better prevent adverse events, halt residents’ worsening health statuses, and avoid the necessity of beginning burdensome treatments to heal pressure ulcers or infections—actions that, in themselves, will save institutions money in the long term.

## 5. Conclusions

To the best of our knowledge, the present systematic review is the first to have integrated longitudinal cohort and interventional studies exploring associations between nursing staff’s working conditions and the QoC given to older adults living in LTRCFs. The review highlighted the predominant influence of human factors on the QoC. Higher overall nursing staff hours worked per resident per day, a suitable number of residents attributed to each caregiver, a reduction in staff turnover, as well as the specific contribution of enough working hours carried out by RNs, along with their special skills and knowledge, can all have a significant positive influence on residents’ clinical outcomes and on results linked to the processes of care. Some organizational elements, such as effective teamwork, more cohesive care teams, and greater levels of nursing autonomy, were all associated with positive impacts on the QoC, whereas other organizational factors, such as assigning permanent or rotating members of staff to residents or the LTRCF’s occupancy rate, only had a relatively small influence on the QoC. Structural factors (such as the size of the LTRCF, whether it was privately or publicly owned, and whether it was located in an urban or rural area) were only weakly associated with the QoC. In the end, it is essential that each LTRCF’s supervisory board, management committee, or decision-making organ makes sure that it promotes optimal working conditions for its nursing staff because these valuable health care professionals have a direct impact on the QoC provided to residents. Particular attention should be given to ensuring that the overall nursing staff’s HPRD is sufficient and that there are enough RNs in the mix of nursing professionals.

## Figures and Tables

**Table 1:** Characteristics of the included studies.

Author Year Country	Study Type	Context	Study Length (Years)	Research Objectives	Methods and Measurement Instruments	Limitations	Recommendations
Hyer et al. [43] (2011), USA	Cohort study	Nursing homes *n* = 663	4	Examine relationships between the HPRDs of CNAs, RNs, and LPNs and the presence of deficiencies	❖ HPRD and facility characteristics: Online Survey, Certification, and Reporting (OSCAR) database and Florida Nursing Home Staffing Reports ❖ Deficiencies: Total deficiency score from CMS’ Nursing Home Compare Five-Star Quality Rating System [44]; quality of care (QoC) deficiency scores [45]	OSCAR’s reliability criticized because HPRD calculated over two weeks only	Increase the number of HPRD for CNAs
Kim et al. [46] (2009), USA	Cohort study	Nursing homes *n* = 1099	5	Examine relationships between HPRDs of CNAs, LPNs, and RNs and the presence of deficiencies	❖ HPRD annual cost report data submitted to the California Office of Statewide Health Planning and Development [47], which includes nursing HPRD (total and individual) and which shows adherence to State staffing standards ❖ Deficiencies: Automated Certification and Licensing Administrative Information and Management System (ACLAIMS) database, which includes Number of total deficiencies (administration, environment, life safety, nutrition, pharmacy, resident rights, QoC, mistreatment, and resident assessment) [48]; QoC deficiencies (QoC, mistreatment, and resident assessment); severe deficiencies that may cause harm or jeopardy	Deficiencies pointed out via occasional inspections	Increase the number of HPRD for nursing staff as a whole, but particularly for RNs
Konetzka et al. [49](2008), USA	Cohort study	Nursing homes *n* = 1366	4	Examine relationships between HPRDs of RNs/skill mix and residents’ health outcomes	❖ Clinical outcomes and case-mix data: Minimum Data Set (MDS) nursing home resident assessment, which includes pressure sores within last 14 days and urinary tract infections (UTIs) within last 30 days ❖ HPRD: Online Survey, Certification, and Reporting (OSCAR) database which includes RN HPRD and skill mix (% of total hours provided by RNs)	OSCAR’s reliability criticized because HPRD calculated over two weeks only	Increase the number of HPRD for RNs and RNs in the skill mix
Kwong et al. [50] (2009), China	Cohort study	Nursing homes *n* = 4	0.83 (10 months)	Evaluate factors affecting the development of pressure ulcers	❖ Pressure ulcer risk form: Chinese version of the MBS [51] and the Braden subscale for nutrition [52] ❖ Human resources form: presence of nurses working in the nursing homes and the proportions of full-time nursing assistants and residents living in the homes	Small sample, including only two LTRCFs with RNs	Ensure a sufficient presence of RNs
Linn et al. [53] (1977), USA	Cohort study	Nursing homes *n* = 30	9	Determine relationships between LTRCF characteristics and outcomes for residents	❖ Facility characteristics: number of beds, bed occupancy rate, waiting lists, staffing hours, staff–patient ratios, total number of staff, and monthly costs; Nursing Home Rating Scale [54] ❖ Residents’ Outcomes: three types of outcome at six months: (a) living or dead; (b) improved, the same, deteriorated, or dead; (c) location: discharged, still in the nursing home, readmitted to the hospital, or dead	Over-representation of LTRCFs from urban areas	Increase the number of HPRD for RNs
Popp et al. [55] (2006), Germany	Cohort study	Nursing homes *n* = 29	0.33 (4 months)	Examine relationships between proportions of qualified personnel and incidence of pressure ulcers	❖ Data source: Hamburger Qualitätsvergleich in der Dekubitusprophylaxe ❖ Proportions of qualified personnel: full-time equivalent posts occupied ❖ Residents classified into three groups: 1) cared for with low (< 50%); 2) medium (50–60%); and 3) high proportions of qualified personnel (≥ 60%) ❖ Incidences of pressure ulcers: number of residents with a pressure ulcer in relation to the total number of residents	Small sample	Carry out studies with larger samples
Shin et al. [56] (2018), South Korea	Cohort study	Nursing homes *n* = 45	2.75 (33 months)	Examine relationships between nursing staff numbers and QoC	❖ Staffing information: collected using “The Nursing Facility Staff Survey” ❖ Facility characteristics: collected from administrators, directors of nursing, and administrative staff (bed size, years in operation, ownership characteristics, chain, religion of the establishment, referral hospitals, location) ❖ Outcomes: 15 indicators of the quality of care from the Korean National Health Insurance Service’s 2015 Nursing Home Evaluation Manual and the U.S. Minimum Data Set [57]	Small sample; high attrition rate; self- reporting methodology	Ensure a sufficient presence of RNs
Temkin et al. [58] (2012), USA	Cohort study	Nursing homes *n* = 162	1.08 (13 months)	Examine associations between work environments and risks of pressure ulcers and incontinence	❖ Environmental attributes: obtained using a survey addressed to all staff members providing direct, daily care (staff cohesion; percentage of staff in self-managed teams or formal teams; percentage of staff with consistent assignment) ❖ Facility characteristics: staffing ratios (RN, LPN, and CNA HPRD); facility location (upstate); facility ownership (not-for-profit, chain membership); percentages of Medicare/Medicaid residents were obtained using the Online Survey Certification and Reporting System (OSCAR) ❖ Outcomes: prevalence of pressure ulcers and urinary/fecal incontinence	Self-reporting methodology	Develop new management strategies (interpersonal communication and coordination of care)
Yoon et al. [59] (2012), South Korea	Cohort study	Long-term care hospitals *n* = 534	0.33 (4 months)	Examine impact of organizational factors on QoC for urinary incontinence	❖ Data sources: Health Insurance Review and Assessment Services review of claims and assessment of care quality ❖ Urinary incontinence (UI) care quality categorized into two groups: (1) Improvement group included residents who experienced improved UI status and remained completely continent. (2) No improvement group included those who deteriorated or did not change their UI status ❖ Organizational characteristics: ownership type, location, operating period, number of beds, nurse staffing level (ratio of total nursing staff, including RNs and LPNs, per 100 beds), and RN ratio (ratio of RNs to total nursing staff)	Self-reporting methodology	Increase the ratio of RNs whatever the overall level of nursing staff
Zimmerman et al. [60] (2018), Germany	Cohort study	Nursing homes *n* = 166	5	Explore differences in nurse staffing levels on resident weight loss	❖ Data Source: EQisA project [61] ❖ Weight loss: percentage of residents per nursing home who had lost more than 10% of their weight within the past 6 months ❖ Staffing: staff-to-resident ratio measured in full-time equivalents ❖ Organizational factors: facility size, bed occupancy rate, location	Majority of LTRCFs belonged to Caritas Association	Further research needed to identify factors leading to weight loss
Burgio et al. [62] (2004), USA	Quasi-experimental study	Nursing homes *n* = 4	10-day periods	Compare QoC results for residents according to permanent or rotating staff assignment to residents and work shifts	❖ Staffing assignment: permanent (PA) and rotating (RA) assignment ❖ Two work shifts: 0700–1500 (morning shift) and 1500–2300 (evening shift) ❖ Data source: collected within each randomly scheduled nursing home from cohorts of eight residents over a 10-day period ❖ Three measures: The computer-assisted behavior observational systems (CABOS), which include two systems (activity time-sampling system and daily care system); The Personal Appearance and Hygiene Index (PAI) [63]; the Affect Rating Scale (ARS) [64] ❖ Outcomes: CNA–resident interaction; resident behavioral disturbances; affect states; personal appearance and hygiene	In LTRCFs with PA of staff, residents were only matched with their primary CNA half of the time	Research is needed to determine impacts of higher rates of staff permanency (> 50%) on residents’ outcomes

**Table 2:** Methodological quality of the cohort studies.

Study	Selection				Comparability		Outcome			Total Quality Score
	Representativeness of Exposed Cohort	Selection of Non-Exposed Cohort	Ascertainment of Exposure	Demonstration That Outcome of Interest Was Not Present at Start of Study	Adjusted for the Most Important Risk Factors	Adjusted for Other Risk Factors	Assessment of Outcome	Follow-Up Length	Loss-to-Follow-Up Rate	
Hyer et al. [43] (2011)	1 *	0 *	1 *	1 *	1 *	1 *	1 *	1 *	0 *	7 *
Linn et al. [53] (1977)	1 *	0 *	1 *	1 *	1 *	1 *	0 *	1 *	1 *	7 *
Kim et al. [46] (2009)	1 *	0 *	1 *	0 *	1 *	1 *	1 *	1 *	0 *	6 *
Kwong et al. [50] (2009)	0 *	0 *	1 *	1 *	1 *	1 *	0 *	1 *	1 *	6 *
Popp et al. [55] (2006)	1 *	1 *	0 *	1 *	1 *	1 *	0 *	1 *	0 *	6 *
Konetzka et al. [49] (2008)	1 *	0 *	1 *	0 *	1 *	1 *	0 *	1 *	0 *	5 *
Yoon et al. [59] (2012)	0 *	0 *	1 *	1 *	1 *	1 *	0 *	1 *	0 *	5 *
Zimmerman et al. [60] (2018)	0 *	0 *	1 *	1 *	1 *	1 *	0 *	1 *	0 *	5 *
Shin et al. [56] (2018)	1 *	0 *	0 *	0 *	1 *	1 *	0 *	1 *	0 *	4 *
Temkin et al. [58] (2012)	0 *	0 *	0 *	0 *	1 *	1 *	0 *	1 *	0 *	3 *

Note: A study could be awarded a maximum of one star for each numbered item in the Selection and Outcome categories. A maximum of two stars could be given for Comparability. Studies were evaluated on a scale from 0 to 9 stars and classified into groups of low (<6 stars), moderate (6–7 stars), or high (8–9 stars) quality. X* = X star.

**Table 3:** Synthesis of the HPRD.

Study	HPRD RN M (SD)	HPRD LPN M (SD)	HPRD CNA M (SD)	HPRD Total RN/LPN/CNA M (SD)
Kim et al. [46] (2009)	0.35 (0.26)	0.61 (0.27)	2.27 (0.41)	3.23 (0.66)
Temkin et al. [58] (2012)	0.61 (0.23)	0.83 (0.25)	2.31 (0.40)	-
Linn et al. [53] (1977)	M = 3.58 (Range = 3.03 to 4.26 *)	M = 0.82 (Range = 0.21 to 1.26 *)	M = 1.14 (Range = 0.04 to 2.53 *)	-
Konetzka et al. [49] (2008)	0.35 (0.22)	-	-	-
Shin et al. [56] (2018)	0.18	0.17 **	2.68 ***	-
Hyer et al. [43] (2011)	1.15 (0.24)		2.49 (0.29)	-

* HPRD data were collected for RNs, LPNs, and CNAs for five distinct groups of residents; ** professionals defined as CNAs in the study; *** professionals defined as QCWs, but equivalent to CNAs.

**Table 4:** Statistical results from the cohort studies.

Authors; Year; Country	Independent Variables (IV)	Dependent Variables (DV)	Covariables	Statistical Results								
				Statistical Analysis	IV	DV	Coefficient	Standard Error	Odds Ratio	Confidence Interval (95%)	*F* Ratio	*p*-Value
Hyer et al. [43] (2011), USA	○ CNA HPRD ○ LPN and RN HPRD	○ Total deficiency score ○ Quality of care (QoC) deficiency scores	Control variables: resident acuity index, number of beds, member of a chain of nursing homes, for-profit facilities, proportion of Medicaid residents and Medicare residents, facility’s occupancy rate, facility’s survey region	Regression models	CNA HPRD	Total deficiency score	−0.10	0.05				* p * = 0.06
					CNA HPRD	QoC deficiency score	−0.29	0.13				* p * = 0.02 *
					LPN–RN HPRD	Total deficiency score	−0.11	0.07				* p * = 0.10
					LPN–RN HPRD	QoC deficiency score	−0.20	0.16				* p * = 0.20
Kim et al. [46] (2009), USA	○ Total nursing HPRD ○ RN HPRD ○ LPN HPRD ○ NA HPRD ○ Meeting state staffing standards	○ Number of total deficiencies ○ Quality of care (QoC) deficiencies ○ Severe deficiencies that may cause harm or jeopardy	Control variables: number of beds, profit status, Medicare-paid days, Medi-Cal-paid days, self-pay days, occupancy rate, nursing home chain affiliation, resident care needs	Poisson random-effects (Res) models	Total nursing HPRD	Total deficiencies	−0.03	0.01				* p * < 0.001 *
					Total nursing HPRD	QoC deficiencies	−0.04	0.01				* p * < 0.001 *
					Total nursing HPRD	Serious deficiencies	−0.10	0.05				* p * < 0.05 *
					RN HPRD	Total deficiencies	−0.07	0.02				* p * < 0.001 *
					RN HPRD	QoC deficiencies	−0.09	0.03				* p * < 0.01 *
					RN HPRD	Serious deficiencies	−0.25	0.13				* p * > 0.05
					LPN HPRD	Total deficiencies	0.12	0.01				* p * < 0.001 *
					LPN HPRD	QoC deficiencies	0.11	0.02				* p * < 0.001 *
					LPN HPRD	Serious deficiencies	0.12	0.11				* p > 0.05 *
					CNA HPRD	Total deficiencies	−0.06	0.01				p < 0.001 *
					CNA HPRD	QoC deficiencies	−0.08	0.02				p < 0.001 *
					CNA HPRD	Serious deficiencies	−0.14	0.07				p < 0.05 *
Konetzka et al. [49] (2008), USA	○ RN HPRD ○ Skill mix (% of total staffing hours, RN, LPN, and NA combined)	○ Pressure ulcers within last 14 days ○ Urinary tract infections (UTIs) within last 30 days	Control variables: proprietary status, Medicare-covered stays, private-pay stays, facility occupancy rate, ADL functioning, index of skilled services, percentage of residents with dementia, depression, psychiatric diagnoses	Fixed effects model with residual inclusion IV	RN HPRD	Pressure ulcers	−3.00	0.52				*p* = 0.01 *
					RN HPRD	UTIs	−1.56	0.41				*p* = 0.01 *
					Skill mix	Pressure ulcers	0.05	0.44				*p* > 0.05
					Skill mix	UTIs	−1.66	0.50				*p* = 0.01 *
					Occupancy rate	Pressure ulcers	−0.04	0.17				*p* > 0.05
					Occupancy rate	UTIs	0.04	0.14				*p* > 0.05
Kwong et al. [50] (2009), China	○ Nurses working in the nursing home (yes) ○ Number of nursing assistants per 100 residents	○ Pressure ulcers developed in last 4 weeks	Control variables: ○ Comorbidities: pneumonia, renal failure, stroke ○ Activity: bedfast, chairfast	Multiple logistic regression	Nurses working in the nursing home (yes)	Pressure ulcers developed in last 4 weeks			0.26	[0.13–0.53]		*p* ≤ 0.001 *
					Number of nursing assistants per 100 residents	Pressure ulcer development in last 4 weeks			1.09	[1.05–1.12]		*p* ≤ 0.001 *
Linn et al. [53] (1977), USA	○ Size ○ RN HPRD ○ LPN HPRD ○ CNA HPRD ○ Total staff/res. ratio ○ Cost/month	Residents were classified by 3 types of outcome, reflecting their status at the end of six months: ○ alive or dead ○ improved, the same, deteriorated, or dead ○ location: discharged, still in the nursing home, readmitted to hospital, or dead.	Control variables: expected outcome, age, cancer, and chronic brain disease	Multivariate analysis of covariance	RN total HPRD	Mortality					4.66	*p* < 0.05 *
						Function					3.03	*p* < 0.05 *
						Location					3.23	*p* < 0.05 *
					LPN total HPRD	Mortality					0.87	*p* > 0.05
						Function					0.21	*p* > 0.05
						Location					1.26	*p* > 0.05
					CNA total HPRD	Mortality					0.04	*p* > 0.05
						Function					2.41	*p* > 0.05
						Location					0.16	*p* > 0.05
					Total staff/resident ratio	Mortality					0.09	*p* > 0.05
						Function					2.68	*p* > 0.05
						Location					0.21	*p* > 0.05
					Size of institution	Mortality					0.10	*p* > 0.05
						Function					2.11	*p* > 0.05
						Location					2.39	*p* > 0.05
					Cost/month	Mortality					0.09	*p* > 0.05
						Function					5.26	p < 0.01*
						Location					0.25	*p* > 0.05
Popp et al. [55] (2006), Germany	○ Care with low (< 50%) proportion of qualified personnel ○ Care with medium proportion (50–60%) ○ Care with high proportion (≥ 60%)	○ Incidence of the development of pressure ulcers	Control variables: ○ Level of nursing care ○ Guidelines on pressure ulcer prophylaxis ○ Specialization in pressure prophylaxis ○ Certified quality management system	Multivariate logistic regression models	Medium proportion (50–60%) of qualified personnel	Incidence of development of a new pressure ulcer			1.50	[0.52–4.35]		*p* = 0.45
					High proportion (≥ 60%) of qualified personnel	Incidence of development of a new pressure ulcer			0.80	[0.25–2.54]		*p* = 0,70
Shin et al. [56] (2018), South Korea	○ Nurse staffing HPRD: RN, LPN, and CNA ○ Skill mix ○ Staff turnover	15 indicators of quality of care:prevalence of falls; pressure score; aggressive behaviors; depression; cognitive decline; incontinence; UTI; weight loss; dehydration; tube feeding; bed rest; ADLs; deteriorated range of motion; antidepressants or sleeping pills; physical restraints	Control variables: ○ Number of beds ○ ownership form (for profit or not) ○ occupancy rate ○ operation duration (years) ○ location (metropolitan / small city / rural) ○ long-term care insurance ○ chain of hospitals (or not) ○ religious establishment (or not)	Repeated measures hierarchical linear model	RN HPRD	Depression	−0.28					*p* = 0.002 *
					RN HPRD	Tube feeding	0.08					*p* = 0.03 *
					RN HPRD	Bed rest	−0.22					*p* = 0.04 *
					LPN HPRD	Physical restraints	−0.04					*p* = 0.01 *
					LPN HPRD	Aggressive behaviors	0.16					*p* < 0.0001 *
					CNA HPRD	Weight loss	0.02					*p* < 0.0001 *
					CNA HPRD	Bed rest	0.05					*p* = 0.005 *
					CNA HPRD	Deteriorated ADLs	0.10					*p* = 0.01 *
					Skill mix (RNs–LPNs)	Aggressive behaviors	−0.05					*p* = 0.03 *
					Skill mix (RNs–LPNs)	Depression	−0.06					*p* = 0.02 *
					Skill mix (RNs–LPNs)	Weight loss	−0.02					*p* = 0.03 *
					Skill mix (RNs–LPNs)	Bed rest	−0.07					*p* = 0.04 *
					Skill mix (RNs–CNAs)	Weight loss	−0.12					*p* = 0.05 *
					RN turnover	Antidepressant	0.01					*p* = 0.00 *
					LPN turnover	Antidepressant	0.01					*p* = 0.02 *
Temkin et al. [58] (2012), USA	○ Staff cohesion ○ Formal teams ○ Self-managed teams ○ Consistent assignment ○ Bed size	○ Prevalence of pressure ulcers ○ Prevalence of urinary/fecal incontinence	Control variables: ○ Staffing ratios (RN, LPN, CNA- HPRD) ○ Facility location (Upstate or downstate) ○ Facility ownership (not-for-profit, chain membership) ○ Percentage of Medicare/ Medicaid residents	Random effects logistic models	Staff cohesion (per 0.23 SD increase)	Pressure ulcers			0.96			*p* = 0.03 *
					Staff cohesion (per 0.23 increase)	Incontinence			0.92			*p* < 0.001 *
					Self-managed teams	Pressure ulcers			0.98			*p* = 0.03 *
					Self-managed teams	Incontinence			0.99			*p* = 0.60
					Primary assignment	Pressure ulcers			1.30			*p* = 0.26
					Primary assignment	Incontinence			0.90			*p* = 0.74
					Bed size	Pressure ulcers			0.10			*p* = 0.56
					Bed size	Incontinence			0.10			*p* = 0.37
					Nursing hours (RN + LPN + CNA)/patient/day)	Pressure ulcers			1.11			*p* = 0.61
					Nursing hours (RN + LPN + CNA)/patient/day)	Incontinence			1.28			*p* = 0.22
Yoon et al. [59] (2012), South Korea	○ Ownership type ○ Location (urban or rural) ○ Operating period ○ Number of beds ○ Doctor staffing level ○ Nurse staffing level (RNs and LPNs per 100 beds) ○ RN ratio	○ Improvement group ○ No improvement group	8 Patients characteristics ○ Sex ○ Age ○ ADL level ○ Cognitive impairment ○ Delirium ○ Depressive mood ○ Stroke ○ Urinary tract infection	Multi-level logistic regression with a random intercept model	Location (urban)	Quality of UI car	0.25	0.11	1.28	[1.03–1.60]		*p* = 0.02*
					Nurse staffing level	Quality of UI care	0.01	0.01	1.01	[0.99–1.04]		*p* = 0.37
					RN ratio	Quality of UI care	0.59	0.26	1.80	[1.08–2.99]		*p* = 0.02 *
					Ownership (private)	Quality of UI care	0.05	0.15	1.05	[0.78–1.40]		*p* = 0.75
					Number of beds	Quality of UI care	0.00	0.00	1.00	[1.00–1.00]		*p* = 0.06
Zimmerman et al. [60] (2018), Germany	○ RN staffing (ratio of residents to RNs) ○ CNA staffing (ratio of residents to CNAs)	○ Weight loss over the past 6 months	Control variables: ○ Location (reference = metropolitan, urban, rural) ○ Region ○ Institution size (number of beds) ○ Occupancy (occupancy rate) ○ Resident case mix ○ Number of residents	Multiple logistic regression	RN staffing	Weight loss			2.30	[1.34–3.93]		*p* ≤ 0.01 *
					NA staffing	Weight loss			0.94	[0.72–1.24]		*p* ≥ 0.05
					Location (urban)	Weight loss			0.77	[0.26–2.1]		*p* ≥ 0.05
					Location (rural)	Weight loss			0.49	[0.17–1.39]		*p* ≥ 0.05
					Institution size	Weight loss			0.99	[0.98–1.01]		*p* ≥ 0.05
					Number of residents	Weight loss			1.09	[1.04–1.16]		*p* ≤ 0.01 *

p * = *p*-value is statistically significant.

**Table 5:** Statistical results from the quasi-experimental study.

Authors (Year) Country	Statistical Analysis	Measures	Independent Variables (IV)	Dependent Variables (DV)	F Statistic with Degree of Freedom F (1186) IV and DV	*p*-value (IV and DV)	Shifts	Mean (M)		Standard Error	F Statistic with Degree of Freedom F (1186) Shifts	*p*-Value (Shifts)
Burgio et al. [62] (2004), USA	Between-groups quasi-experimental comparison design: Repeated measures analyses of variance	Direct observational systems activity time-sampling system	Rotating assignment (RA) staffing	Resident–CNA spoken interaction (occurrence per 5 min interval)	-	*p* > 0.05	a.m. shift	0.50		0.90	-	*p* > 0.05
							p.m. shift	0.79		1.34		
				CNA–resident interaction (% occurrence overall)	-	*p* > 0.05	a.m. shift	2.57		4.77	-	*p* > 0.05
							p.m. shift	2.66		4.03		
				Resident disruptive behavior (% occurrence overall)	-	*p* > 0.05	a.m. shift	4.50		11.06	10.83	*p* = 0.001 *
							p.m. shift	7.38		17.36		
			Permanent assignment (PA) staffing	Resident–CNA spoken interaction (occurrence per 5 min interval)	-	*p* > 0.05	a.m. shift	0.46		1.03	-	*p* > 0.05
							p.m. shift	0.67		1.08		
				CNA–resident interaction (% occurrence overall)	-	*p* > 0.05	a.m. shift	2.70		5.70	-	*p* > 0.05
							p.m. shift	3.17		4.74		
				Resident disruptive behavior (% occurrence overall)	-	*p* > 0.05	a.m. shift	3.87		10.28	10.83	*p* = 0.001 *
							p.m. shift	7.06		14.59		
		Direct Observational Systems: daily care system	Rotating assignment (RA) staffing	Resident–CNA non-negative spoken interaction (% occurrence overall)	-	*p* > 0.05	a.m. shift	0.70		0.68	4.37	*p* = 0.03 *
							p.m. shift	1.02		1.11		
				CNA–resident task-related positive spoken interaction (% occurrence overall)	-	*p* > 0.05	a.m. shift	74.62		31.27	-	*p* > 0.05
							p.m. shift	79.29		30.83		
				Resident disruptive behavior (% occurrence overall)	-	*p* > 0.05	a.m. shift	12.13		23.46	-	*p* > 0.05
							p.m. shift	10.12		24.32		
			Permanent assignment (PA) staffing	Resident–CNA nonnegative verbal interaction (% occurrence overall)	-	*p* > 0.05	a.m. shift	0.56		0.70	4.37	*p* = 0.03 *
							p.m. shift	0.76		0.94		
				CAN-resident task-related positive verbal interaction (% occurrence overall)	-	*p* > 0.05	a.m. shift	78.88		29.27	-	*p* > 0.05
							p.m. shift	81.03		27.48		
				Resident disruptive behavior (% occurrence overall)	-	*p* > 0.05	a.m. shift	10.60		21.02	-	*p* > 0.05
							p.m. shift	9.19		19.51		
		Paper-and-Pencil Measures: The Personal Appearance and Hygiene Index (PAI)	Rotating assignment (RA) staffing	Staff rating of residents’ personal appearance and hygiene	3.94	*p* = 0.04 *	a.m. shift	87.10		7.10	5.70	*p* = 0.01 *
							p.m. shift	84.80		7.70		
			Permanent assignment (PA) staffing				a.m. shift	87.40		7.90		
							p.m. shift	86.80		7.40		
		Affect Rating Scale (ARS)	Rotating assignment (RA) staffing	Amount of time for which residents expressed any of the affect states	-	*p* > 0.05	a.m. shift	Interest	94.20	17.10	15.71	*p* = 0.0001 *
							p.m. shift		94.50	14.70		
			Permanent assignment (PA) staffing				a.m. shift		97.40	10.00		
							p.m. shift		89.20	25.30		

*p* * = *p*-value is statistically significant.

## Appendix A

Bibliographic database search strategies

Note: the research strategies were peer reviewed by another information specialist prior to execution.

Embase.com

Accessed on 22 May 2020

1508 references found

(‘residential home’/de OR ‘nursing home’/de OR ‘residential care’/de OR ‘home for the aged’/de OR (“residential facilit*” OR “residential home*” OR “residential institution*” OR “residential care” OR “residence care” OR “assisted living facilit*” OR “nursing home*” OR “skilled nursing facilit*” OR “home$ for the aged” OR “old age home” OR “old people home” OR “long-term care facilit*” OR “care homes” OR “long-term care setting*”):ab,ti,kw) AND (‘aged’/exp OR ‘elderly care’/de OR ‘geriatric care’/exp OR ‘geriatric patient’/de OR ‘geriatrics’/exp OR (elder* OR eldest OR geriatr* OR “old age*” OR (older NEXT/1 (patient* OR people OR subject* OR age* OR adult* OR man OR men OR woman OR women OR population* OR person*)) OR aging OR ageing OR senior* OR “late life” OR “oldest old*” OR “very old*”):ab,ti,kw) AND (‘nursing staff’/de OR ‘nurse’/exp OR ‘nursing’/exp OR (nursing OR nurse OR nurses):ab,ti,kw) AND (‘health care quality’/de OR ‘clinical effectiveness’/de OR ‘clinical indicator’/de OR ‘health care survey’/de OR ‘incident report’/de OR ‘medication error’/exp OR ‘near miss (health care)’/de OR ‘nursing outcome’/de OR ‘quality of nursing care’/de OR ‘treatment outcome’/exp OR ‘quality control’/de OR ‘nursing audit’/de OR ‘total quality management’/de OR ((quality NEAR/4 (care OR healthcare OR “health care” OR nursing)) OR (evaluation NEAR/3 (care OR healthcare OR “health care”)) OR “standard of care” OR (quality NEAR/3 indicator*) OR “health metric*” OR (outcome* NEAR/3 (assessment OR treatment* OR nursing)) OR (clinical NEXT/1 (effectiveness OR indicator*)) OR ((care OR healthcare OR “health care”) NEXT/1 survey*) OR “incident report*” OR “medication error*” OR “quality management” OR “quality control*” OR “nursing audit*”):ab,ti,kw) AND (‘work’/exp OR ‘workforce’/exp OR ‘occupation’/exp OR ‘occupational health’/exp OR ((work* NEXT/3 (condition* OR capacity OR environment OR engagement OR experience OR performance OR schedul* OR satisfaction* OR stress*)) OR workforce* OR “work force*” OR workplace* OR “labor force*” OR “labour force*” OR manpower OR “work load” OR workload* OR “work load*” OR “working load” OR absenteeism OR presenteeism OR burnout OR burn-out OR turnover OR “occupational stress” OR “compassion fatigue” OR “personnel management” OR work-life OR “working life” OR occupation OR career OR employment OR job OR profession* OR vocation* OR staffing OR “occupational health”):ab,ti,kw)

Medline Ovid SP

Ovid MEDLINE(R) and Epub Ahead of Print, In-Process & Other Non-Indexed Citations and Daily 1946 to May 21, 2020

Accessed on 22 May 2020 1106 references found

(“Residential Facilities”/ OR “Assisted Living Facilities”/ OR “Homes for the Aged”/ OR “Nursing Homes”/ OR (“residential facilit*” OR “residential home*” OR “residential institution*” OR “residential care” OR “residence care” OR “assisted living facilit*” OR “nursing home*” OR “skilled nursing facilit*” OR “home? for the aged” OR “old age home” OR “old people home” OR “long-term care facilit*” OR “care homes” OR “long-term care setting*”).ab,ti,kf.) AND (exp “Aged”/ OR “Geriatric Nursing”/ OR “Geriatrics”/ OR (elder* OR eldest OR geriatr* OR “old age*” OR (older ADJ1 (patient* OR people OR subject* OR age* OR adult* OR man OR men OR woman OR women OR population* OR person*)) OR aging OR ageing OR senior* OR “late life” OR “oldest old*” OR “very old*”).ab,ti,kf.) AND (Exp “Nursing Staff”/ OR exp “Nurses”/ OR exp “Nursing”/ OR “Nurse’s Role”/ OR “nursing”.fs. OR (nursing OR nurse OR nurses).ab,ti,kf.) AND (“Quality of Health Care”/ OR exp “Outcome and Process Assessment, Health Care”/ OR exp “Quality Indicators, Health Care”/ OR “Quality Control”/ OR exp “Medication Errors” OR “Nursing Audit”/ OR “Total Quality Management”/ OR ((quality ADJ4 (care OR healthcare OR “health care” OR nursing)) OR (evaluation ADJ3 (care OR healthcare OR “health care”)) OR “standard of care” OR (quality ADJ3 indicator*) OR “health metric*” OR (outcome* ADJ3 (assessment OR treatment* OR nursing)) OR (clinical ADJ1 (effectiveness OR indicator*)) OR ((care OR healthcare OR “health care”) ADJ1 survey*) OR “incident report*” OR “medication error*” OR “quality management” OR “quality control*” OR “nursing audit*”).ab,ti,kf.) AND (exp “Work”/ OR exp “Workforce”/ OR exp “Occupations”/ OR “Health Occupations”/ OR exp “Personnel Management”/ OR “Occupational Health”/ OR “Job Satisfaction”/ OR ((work* ADJ3 (condition* OR capacity OR environment OR engagement OR experience OR performance OR schedul* OR satisfaction* OR stress*)) OR workforce* OR “work force*” OR workplace* OR “labor force*” OR “labour force*” OR manpower OR “work load” OR workload* OR “work load*” OR “working load” OR absenteeism OR presenteeism OR burnout OR burn-out OR turnover OR “occupational stress” OR “compassion fatigue” OR “personnel management” OR work-life OR “working life” OR occupation OR career OR employment OR job OR profession* OR vocation* OR staffing OR “occupational health”).ab,ti,kf.)

PubMed

Accessed on 22 May 2020

186 references found

Limit: NOT medline[sb]

(“residential facilit*”[tiab] OR “residential home*”[tiab] OR “residential institution*”[tiab] OR “residential care”[tiab] OR “residence care”[tiab] OR “assisted living facilit*”[tiab] OR “nursing home*”[tiab] OR “skilled nursing facilit*”[tiab] OR “home for the aged”[tiab] OR “homes for the aged”[tiab] OR “old age home”[tiab] OR “old people home”[tiab] OR “long-term care facilit*”[tiab] OR “care homes”[tiab] OR “long-term care setting*”[tiab]) AND (elder*[tiab] OR eldest[tiab] OR geriatr*[tiab] OR “old age*”[tiab] OR (older[tiab] AND (patient*[tiab] OR people[tiab] OR subject*[tiab] OR age*[tiab] OR adult*[tiab] OR man[tiab] OR men[tiab] OR woman[tiab] OR women[tiab] OR population*[tiab] OR person*[tiab])) OR aging[tiab] OR ageing[tiab] OR senior*[tiab] OR “late life”[tiab] OR “oldest old*”[tiab] OR “very old*”[tiab]) AND (nursing[tiab] OR nurse[tiab] OR nurses[tiab]) AND ((quality[tiab] AND (care[tiab] OR healthcare[tiab] OR “health care”[tiab] OR nursing[tiab])) OR (evaluation[tiab] AND (care[tiab] OR healthcare[tiab] OR “health care”[tiab])) OR “standard of care”[tiab] OR (quality[tiab] AND indicator*[tiab]) OR “health metric*”[tiab] OR (outcome*[tiab] AND (assessment[tiab] OR treatment*[tiab] OR nursing[tiab])) OR (clinical[tiab] AND (effectiveness[tiab] OR indicator*[tiab])) OR ((care[tiab] OR healthcare[tiab] OR “health care”[tiab]) AND survey*[tiab]) OR “incident report*”[tiab] OR “medication error*”[tiab] OR “quality management”[tiab] OR “quality control*”[tiab] OR “nursing audit*”[tiab]) AND ((work*[tiab] AND (condition*[tiab] OR capacity[tiab] OR environment[tiab] OR engagement[tiab] OR experience[tiab] OR performance[tiab] OR schedul*[tiab] OR satisfaction*[tiab] OR stress*[tiab])) OR workforce*[tiab] OR “work force*”[tiab] OR workplace*[tiab] OR “labor force*”[tiab] OR “labour force*”[tiab] OR manpower[tiab] OR “work load”[tiab] OR workload*[tiab] OR “work load*”[tiab] OR “working load”[tiab] OR absenteeism[tiab] OR presenteeism[tiab] OR burnout[tiab] OR burn-out[tiab] OR turnover[tiab] OR “occupational stress”[tiab] OR “compassion fatigue”[tiab] OR “personnel management”[tiab] OR work-life[tiab] OR “working life”[tiab] OR occupation[tiab] OR career[tiab] OR employment[tiab] OR job[tiab] OR profession*[tiab] OR vocation*[tiab] OR staffing[tiab] OR “occupational health”[tiab]) NOT medline[sb]

CINAHL EBSCO

Accessed on 22 May 2020

1468 references found

(MH “Nursing Homes” OR MH “Nursing Home Patients” OR MH “Residential Care” OR TI (“residential facilit*” OR “residential home*” OR “residential institution*” OR “residential care” OR “residence care” OR “assisted living facilit*” OR “nursing home*” OR “skilled nursing facilit*” OR “home# for the aged” OR “old age home” OR “old people home” OR “long-term care facilit*” OR “care homes” OR “long-term care setting*”) OR AB (“residential facilit*” OR “residential home*” OR “residential institution*” OR “residential care” OR “residence care” OR “assisted living facilit*” OR “nursing home*” OR “skilled nursing facilit*” OR “home# for the aged” OR “old age home” OR “old people home” OR “long-term care facilit*” OR “care homes” OR “long-term care setting*”)) AND (MH “Aged+” OR MH “Gerontologic Care” OR MH “Geriatrics” OR TI (elder* OR eldest OR geriatr* OR “old age*” OR (older W0 (patient* OR people OR subject* OR age* OR adult* OR man OR men OR woman OR women OR population* OR person*)) OR aging OR ageing OR senior* OR “late life” OR “oldest old*” OR “very old*”) OR AB (elder* OR eldest OR geriatr* OR “old age*” OR (older W0 (patient* OR people OR subject* OR age* OR adult* OR man OR men OR woman OR women OR population* OR person*)) OR aging OR ageing OR senior* OR “late life” OR “oldest old*” OR “very old*”)) AND (MH “Nursing Home Personnel” OR MH “Nurses+” OR MH “Nursing Staff, Hospital” OR TI (nursing OR nurse OR nurses) OR AB (nursing OR nurse OR nurses)) AND (MH “Quality of Health Care+” OR MH “Health Care Errors+” OR MH “Medication Errors+” OR MH “Incident Reports” OR MH “Nursing Outcomes” OR MH “Nursing Assessment” OR MH “Quality Control (Technology)” OR TI ((quality N3 (care OR healthcare OR “health care” OR nursing)) OR (evaluation N2 (care OR healthcare OR “health care”)) OR “standard of care” OR (quality N2 indicator*) OR “health metric*” OR (outcome* N2 (assessment OR treatment* OR nursing)) OR (clinical W0 (effectiveness OR indicator*)) OR ((care OR healthcare OR “health care”) W0 survey*) OR “incident report*” OR “medication error*” OR “quality management” OR “quality control*” OR “nursing audit*”) OR AB ((quality N3 (care OR healthcare OR “health care” OR nursing)) OR (evaluation N2 (care OR healthcare OR “health care”)) OR “standard of care” OR (quality N2 indicator*) OR “health metric*” OR (outcome* N2 (assessment OR treatment* OR nursing)) OR (clinical W0 (effectiveness OR indicator*)) OR ((care OR healthcare OR “health care”) W0 survey*) OR “incident report*” OR “medication error*” OR “quality management” OR “quality control*” OR “nursing audit*”)) AND (MH “Work+” OR MH “Work Environment+” OR MH “Workforce” OR MH “Occupations and Professions+” OR MH “Employment+” OR MH “Personnel Management+” OR MH “Occupational Health+” OR MH “Psychology, Occupational+” OR TI ((work* W2 (condition* OR capacity OR environment OR engagement OR experience OR performance OR schedul* OR satisfaction* OR stress*)) OR workforce* OR “work force*” OR workplace* OR “labor force*” OR “labour force*” OR manpower OR “work load” OR workload* OR “work load*” OR “working load” OR absenteeism OR presenteeism OR burnout OR burn-out OR turnover OR “occupational stress” OR “compassion fatigue” OR “personnel management” OR work-life OR “working life” OR occupation OR career OR employment OR job OR profession* OR vocation* OR staffing OR “occupational health”) OR AB ((work* W2 (condition* OR capacity OR environment OR engagement OR experience OR performance OR schedul* OR satisfaction* OR stress*)) OR workforce* OR “work force*” OR workplace* OR “labor force*” OR “labour force*” OR manpower OR “work load” OR workload* OR “work load*” OR “working load” OR absenteeism OR presenteeism OR burnout OR burn-out OR turnover OR “occupational stress” OR “compassion fatigue” OR “personnel management” OR work-life OR “working life” OR occupation OR career OR employment OR job OR profession* OR vocation* OR staffing OR “occupational health”))

APA PsycINFO OVID SP

APA PsycInfo 1806 to May Week 3 2020

Accessed on 22 May 2020

517 references found

(residential care institutions/ OR nursing homes/ OR (“residential facilit*” OR “residential home*” OR “residential institution*” OR “residential care” OR “residence care” OR “assisted living facilit*” OR “nursing home*” OR “skilled nursing facilit*” OR “home? for the aged” OR “old age home” OR “old people home” OR “long-term care facilit*” OR “care homes” OR “long-term care setting*”).mp.) AND (geriatric patients/ OR exp geriatrics/ OR exp aging/ OR (elder* OR eldest OR geriatr* OR “old age*” OR (older ADJ1 (patient* OR people OR subject* OR age* OR adult* OR man OR men OR woman OR women OR population* OR person*)) OR aging OR ageing OR senior* OR “late life” OR “oldest old*” OR “very old*”).mp.) AND (exp nurses/ OR nursing/ OR (nursing OR nurse OR nurses).mp.) AND (“quality of care”/ OR exp organizational effectiveness/ OR exp treatment outcomes/ OR ((quality ADJ4 (care OR healthcare OR “health care” OR nursing)) OR (evaluation ADJ3 (care OR healthcare OR “health care”)) OR “standard of care” OR (quality ADJ3 indicator*) OR “health metric*” OR (outcome* ADJ3 (assessment OR treatment* OR nursing)) OR (clinical ADJ1 (effectiveness OR indicator*)) OR ((care OR healthcare OR “health care”) ADJ1 survey*) OR “incident report*” OR “medication error*” OR “quality management” OR “quality control*” OR “nursing audit*”).mp.) AND (exp working conditions/ OR “quality of work life”/ OR work load/ OR occupational stress/ OR job satisfaction/ OR exp occupations/ OR exp employment status/ OR exp occupational health/ OR ((work* ADJ3 (condition* OR capacity OR environment OR engagement OR experience OR performance OR schedul* OR satisfaction* OR stress*)) OR workforce* OR “work force*” OR workplace* OR “labor force*” OR “labour force*” OR manpower OR “work load” OR workload* OR “work load*” OR “working load” OR absenteeism OR presenteeism OR burnout OR burn-out OR turnover OR “occupational stress” OR “compassion fatigue” OR “personnel management” OR work-life OR “working life” OR occupation OR career OR employment OR job OR profession* OR vocation* OR staffing OR “occupational health”).mp.)

Cochrane Library Wiley

Cochrane Database of Systematic Reviews Issue 5 of 12, May 2020

Accessed on 22 May 2020

98 references found

(“residential facilit*” OR “residential home*” OR “residential institution*” OR “residential care” OR “residence care” OR “assisted living facilit*” OR “nursing home*” OR “skilled nursing facilit*” OR “home? for the aged” OR “old age home” OR “old people home” OR “long-term care facilit*” OR “care homes” OR “long-term care setting*”):ab,ti,kw AND (elder* OR eldest OR geriatr* OR “old age*” OR (older NEXT/1 (patient* OR people OR subject* OR age* OR adult* OR man OR men OR woman OR women OR population* OR person*)) OR aging OR ageing OR senior* OR “late life” OR “oldest old*” OR “very old*”):ab,ti,kw AND (nursing OR nurse OR nurses):ab,ti,kw AND ((quality NEAR/4 (care OR healthcare OR “health care” OR nursing)) OR (evaluation NEAR/3 (care OR healthcare OR “health care”)) OR “standard of care” OR (quality NEAR/3 indicator*) OR “health metric*” OR (outcome* NEAR/3 (assessment OR treatment* OR nursing)) OR (clinical NEXT/1 (effectiveness OR indicator*)) OR ((care OR healthcare OR “health care”) NEXT/1 survey*) OR “incident report*” OR “medication error*” OR “quality management” OR “quality control*” OR “nursing audit*”):ab,ti,kw AND ((work* NEXT/3 (condition* OR capacity OR environment OR engagement OR experience OR performance OR schedul* OR satisfaction* OR stress*)) OR workforce* OR “work force*” OR workplace* OR “labor force*” OR “labour force*” OR manpower OR “work load” OR workload* OR “work load*” OR “working load” OR absenteeism OR presenteeism OR burnout OR burn-out OR turnover OR “occupational stress” OR “compassion fatigue” OR “personnel management” OR work-life OR “working life” OR occupation OR career OR employment OR job OR profession* OR vocation* OR staffing OR “occupational health”):ab,ti,kw

Web Of Science—Core Collection

Accessed on 22 May 2020

586 references found

TS=((“residential facilit*” OR “residential home*” OR “residential institution*” OR “residential care” OR “residence care” OR “assisted living facilit*” OR “nursing home*” OR “skilled nursing facilit*” OR “home? for the aged” OR “old age home” OR “old people home” OR “long-term care facilit*” OR “care homes” OR “long-term care setting*”) AND (elder* OR “eldest” OR geriatr* OR “old age*” OR (older NEAR/1 (patient* OR “people” OR subject* OR age* OR adult* OR “man” OR “men” OR “woman” OR “women” OR population* OR person*)) OR “aging” OR “ageing” OR senior* OR “late life” OR “oldest old*” OR “very old*”) AND (“nursing” OR “nurse” OR “nurses”) AND ((“quality” NEAR/4 (“care” OR “healthcare” OR “health care” OR “nursing”)) OR (“evaluation” NEAR/3 (“care” OR “healthcare” OR “health care”)) OR “standard of care” OR (“quality” NEAR/3 indicator*) OR “health metric*” OR (outcome* NEAR/3 (“assessment” OR treatment* OR “nursing”)) OR (“clinical” NEAR/1 (“effectiveness” OR indicator*)) OR ((“care” OR “healthcare” OR “health care”) NEAR/1 survey*) OR “incident report*” OR “medication error*” OR “quality management” OR “quality control*” OR “nursing audit*”) AND ((work* NEAR/3 (condition* OR “capacity” OR “environment” OR “engagement” OR “experience” OR “performance” OR schedul* OR satisfaction* OR stress*)) OR workforce* OR “work force*” OR workplace* OR “labor force*” OR “labour force*” OR “manpower” OR “work load” OR workload* OR “work load*” OR “working load” OR “absenteeism” OR “presenteeism” OR “burnout” OR “burn-out” OR “turnover” OR “occupational stress” OR “compassion fatigue” OR “personnel management” OR “work-life” OR “working life” OR “occupation” OR “career” OR “employment” OR “job” OR profession* OR vocation* OR “staffing” OR “occupational health”))

Joanna Briggs Institute EBP Database OVID SP

Joanna Briggs Institute EBP Database-Current to May 13, 2020

Accessed on 22 May 2020

14 references found

(“residential facilit*” OR “residential home*” OR “residential institution*” OR “residential care” OR “residence care” OR “assisted living facilit*” OR “nursing home*” OR “skilled nursing facilit*” OR “home? for the aged” OR “old age home” OR “old people home” OR “long-term care facilit*” OR “care homes” OR “long-term care setting*”) AND (elder* OR eldest OR geriatr* OR “old age*” OR (older ADJ1 (patient* OR people OR subject* OR age* OR adult* OR man OR men OR woman OR women OR population* OR person*)) OR aging OR ageing OR senior* OR “late life” OR “oldest old*” OR “very old*”) AND (nursing OR nurse OR nurses).ti,hw. AND ((quality ADJ4 (care OR healthcare OR “health care” OR nursing)) OR (evaluation ADJ3 (care OR healthcare OR “health care”)) OR “standard of care” OR (quality ADJ3 indicator*) OR “health metric*” OR (outcome* ADJ3 (assessment OR treatment* OR nursing)) OR (clinical ADJ1 (effectiveness OR indicator*)) OR ((care OR healthcare OR “health care”) ADJ1 survey*) OR “incident report*” OR “medication error*” OR “quality management” OR “quality control*” OR “nursing audit*”) AND ((work* ADJ3 (condition* OR capacity OR environment OR engagement OR experience OR performance OR schedul* OR satisfaction* OR stress*)) OR workforce* OR “work force*” OR workplace* OR “labor force*” OR “labour force*” OR manpower OR “work load” OR workload* OR “work load*” OR “working load” OR absenteeism OR presenteeism OR burnout OR burn-out OR turnover OR “occupational stress” OR “compassion fatigue” OR “personnel management” OR work-life OR “working life” OR occupation OR career OR employment OR job OR profession* OR vocation* OR staffing OR “occupational health”).ti,hw.
